# Thyme essential oil nanoemulsion enhances fluconazole efficacy in resistant *Candida glabrata* and *Candida krusei*

**DOI:** 10.22034/cmm.2025.345402.1656

**Published:** 2026-01-01

**Authors:** Donya Nikaein, Seyed Mehdi Joghataei, Javad Malakootikhah, Alireza Shahabi, Erfan Alihosseini, Alireza Khosravi

**Affiliations:** 1 Department of Microbiology and Immunology, Faculty of Veterinary Medicine, University of Tehran, Tehran, Iran; 2 Researcher and Ph.D. graduate in Nanobiotechnology, University of Tehran, Tehran, Iran

**Keywords:** *Candida*, Fluconazole, Nanoemulsion, Resistance gene, Thyme

## Abstract

**Background and Purpose:**

Candidiasis is a common fungal infection, with increasing cases caused by resistant non-albicans species, such as *Candida krusei* and *Candida glabrata*. These species present remarkable clinical treatment challenges due to their resistance, particularly with azole antifungals, like fluconazole (FLZ). This study aimed to evaluate the antifungal potential of thyme (*Thymus vulgaris*) essential oil nanoemulsion (TEONE) against these resistant *Candida* strains, focusing on its impact on resistance gene expression and the enhancement of conventional treatment efficacy.

**Materials and Methods:**

TEONE was formulated by combining thyme EO with Tween 80 and water using the antisolvent precipitation method to create a stable nanoemulsion. The TEON was characterized by using Scanning Electron Microscopy for morphology, dynamic light scattering for particle size, and zeta potential for stability and surface charge assessment. The antifungal activity of TEONE, Thyme EO, and FLZ was assessed against *Candida* strains using disk diffusion, microdilution, and checkerboard assays. The quantitative reverse transcription polymerase chain reaction was employed to evaluate the expression of resistance genes (*ABC1*, *CDR1*, and *ERG11*) following treatment.

**Results:**

The Scanning Electron Microscopy revealed a predominantly spherical morphology with minimal rod-shaped particles, while Dynamic Light Scattering confirmed a mean droplet size of 134.3 nm and a polydispersity index of 0.402, indicating good dispersion. Zeta potential analysis showed a value of -78.6 mV, suggesting strong stability of nanoemulsion. The TEONE demonstrated significant antifungal activity, reducing minimum inhibitory concentration values by up to 50%, compared to Thyme EO (*p*<0.05). Disk diffusion tests showed larger inhibition zones for TEONE (20-25 mm), compared to Thyme EO (15-18 mm) (*p*<0.01). The quantitative reverse transcription polymerase chain reaction revealed that TEONE reduced CgCDR1 expression by up to 49% in *Candida glabrata* and downregulated *ERG11* and *ABC1* in *Candida krusei* (*p*<0.05).

**Conclusion:**

While FLZ alone tended to increase resistance gene expression, combining TEONE with FLZ mitigated this effect and enhanced antifungal efficacy, indicating an additive interaction. TEONE represents a promising natural approach for enhancing antifungal treatment strategies against resistant *Candida* infections, offering potential benefits as an alternative or complementary therapy to conventional antifungal agents. These nanoemulsions may also allow for a reduction in the dosage of traditional drugs such as FLZ, potentially minimizing side effects and the development of resistance.

## Introduction

Candidiasis is a widespread fungal infection caused by various species of the *Candida* genus, which can affect the mucosal surfaces, skin, and even internal organs, leading to remarkable morbidity and mortality, especially among immunocompromised individuals [1-3]. Among these species, *Candida albicans* remains the most common pathogen; however, non-*albicans* species, such as *Candida krusei* and *Candida glabrata*, have been increasingly reported, especially in nosocomial (hospital-acquired) infections [4-6]. These non-albicans species are often associated with higher rates of antifungal resistance, posing significant challenges for treatment [7, 8]. The rise in resistance is particularly alarming in cases involving azole antifungals, like fluconazole (FLZ), which are commonly used in the management of candidiasis [9].

Azoles, such as FLZ, itraconazole, posaconazole, and voriconazole (VRC), are among the most commonly used antifungal agents for treating candidiasis. Resistance to azoles in *Candida* species typically involves changes in the target enzyme 14α-lanosterol demethylase (14-DM), encoded by the *ERG11* gene, as well as the upregulation of efflux pumps, including multidrug resistance proteins [10, 11]. *ERG11* mutations or overexpression have been linked to resistance in *C. albicans*, *C. glabrata*, and *C. tropicalis*. Efflux pumps associated with resistance include members of the ATP-binding cassette transporter family, such as *CDR1*, *CDR2*, and *MDR1* in *C. albicans* and *CDR1*, *CDR2*, and *SNQ2* in *C. glabrata*.

While research has predominantly focused on *C. albicans*, *C. glabrata*, and *C. tropicalis*, studies on *C. krusei* resistance are limited. *Candida krusei* is naturally resistant to FLZ, primarily due to reduced susceptibility of 14-DM to the drug and the role of the efflux pump gene *ABC1* [12-17].

The growing issue of antifungal resistance necessitates the development of novel treatment strategies. One promising approach is using natural antifungal agents, such as essential oils (EOs), which have been recognized for their antimicrobial properties [18]. EOs like Thyme oil (*Thymus vulgaris*) have shown significant potential due to their broad-spectrum activity against various fungal pathogens, including azole-resistant *Candida* strains [19]. Recent advances in nanotechnology have led to the development of nanoemulsion formulations of EOs, enhancing their stability, bioavailability, and antifungal efficacy [20-22]. Nanoemulsions can improve the delivery and penetration of active compounds into fungal cells, overcoming some of the limitations associated with conventional antifungal therapies [23, 24].

In addition to enhancing antifungal efficacy, combining thyme (*Thymus vulgaris*) essential oil nanoemulsion (TEONE) with FLZ may offer important clinical benefits by enabling dose reduction of FLZ without compromising therapeutic outcomes [25, 26]. Lowering FLZ dosages can help minimize adverse effects, such as hepatotoxicity and drug–drug interactions, which are especially critical in immunocompromised patients who often require complex medication regimens. Moreover, reducing the antifungal burden may help slow the development of resistance, thereby extending the clinical utility of existing azole therapies [27, 28].

Thyme EO is known for its potent antifungal properties, attributed primarily to its high content of bioactive compounds, like Thymol and Carvacrol [29, 30]. These compounds disrupt the cell membranes of fungi, leading to cell lysis and death, effectively inhibiting the growth of various pathogenic fungi, including *Candida* species [31]. Studies have shown that Thyme oil exhibits broad-spectrum antifungal activity against both azole-resistant and susceptible fungal strains, making it a valuable natural alternative for managing fungal infections [32]. Furthermore, incorporating Thyme oil into nanoemulsion formulations has enhanced its stability, bioavailability, and efficacy, thereby improving its therapeutic potential [33].

This study aimed to investigate the antifungal efficacy of TEONE against *C. krusei* and *C. glabrata*, mainly focusing on strains that exhibit resistance to FLZ. Previous research has shown that nanoemulsions can significantly enhance the activity of EOs by reducing the minimum inhibitory concentrations (MICs) (μg/ml) required to inhibit fungal growth [34].

In addition, nanoemulsion-based formulations have demonstrated the ability to downregulate key resistance genes, such as *ABC1*, *CgCDR1*, and *ERG11*, thereby potentially reversing resistance mechanisms and restoring the efficacy of conventional antifungal drugs [14, 15, 35, 36]. However, to date, no study has explicitly examined the synergistic potential of TEONE and FLZ against *C. krusei* and *C. glabrata*, representing a significant gap in the literature. By exploring the effectiveness of TEONE in conjunction with FLZ, this study aimed to provide insights into alternative therapeutic strategies for managing resistant *Candida* infections.

## Materials and Methods

### Materials

Thyme EO, extracted from *T. vulgaris*, was sourced from the Iranian Institute of Medicinal Plants in Iran (product approval number: PCA-13; request code: 0105-0021). Based on the GC/MS analysis results by the manufacturer, the major Thyme EO compounds identified in the Iranian Institute of Medicinal Plants were Thymol (36.74%), Borneol (11.36%), para-Cymene (10.09%), linalool (3.46 %), alpha-Pinene (4.29%), and Carvacrol (5.07%). Tween 80 (polyoxyethylene [20] sorbitan monooleate), a non-ionic surfactant utilized to prepare the nanoemulsion, was obtained from Fisher Scientific, Geel, Belgium. The culture media necessary for fungal growth and testing were procured from Merck, Germany. Sterile blank paper discs (6 mm in diameter) were obtained from Padtan-Teb Company, Iran, for disk diffusion assays. The FLZ discs were acquired in a concentration of 25 μg from Thermo Scientific^TM^, USA. The FLZ powders were acquired from Sigma-Aldrich, St. Louis, MO, USA.

Distilled water was employed throughout all experiments, ensuring purity and consistency in preparing solutions and formulations. All chemicals and reagents used in this study were of analytical grade, guaranteeing the reliability and accuracy of the experimental results.

### Fabrication of nanoemulsion thyme essential oil

This study formulated TEONE using Thyme EO, a non-ionic surfactant (Tween 80), and water using the antisolvent precipitation method [33]. The preparation began by creating a composite emulsifier. To accomplish this, 20 mL of Thyme EO was gradually combined with 5 mL of Tween 80 (surfactant) under gentle stirring conditions (FJ200-S; Lichen Instrument Technology Co., Ltd, Hunan, China) to create a uniform mixture. After the emulsifier was formed, 75 mL of water was slowly incorporated into the blend, bringing the total volume to 100 mL.

The prepared mixture was then subjected to magnetic stirring at 10,000 rpm for 15 min to ensure complete homogenization. Following this initial mixing step, the mixture was treated with sonication using an ultrasonicator (Scientz-II D; Ningbo Scientz, Zhejiang, China) for 20 min, operating at a power level of 450 W, which represents 50% of the total input power of the device (950 W). During the sonication process, the nanoemulsion samples were kept in an ice bath to prevent overheating and maintain their stability.

The nanoemulsion was prepared in different formulations by optimizing the surfactant concentration while keeping the EO concentration constant. The various nanoemulsion formulations were prepared at different concentrations, specifically at 5,000, 2,500, 1,250, and 625 ppm. The nanoemulsion formulations were prepared by maintaining the Thyme EO concentrations at 5,000 ppm (500 mg of EO in 100 mL of the nanoemulsion), 2,500 ppm (250 mg of EO in 100 mL), 1,250 ppm (125 mg of EO in 100 mL), and 625 ppm (62.5 mg of EO in 100 mL), ensuring consistent EO levels across all formulations for comparative analysis. In order to standardize the calculated dose, the required amount of Thyme EO for each concentration was precisely weighed using an analytical balance (±0.1 mg accuracy), and the total volume of the mixture was adjusted to 100 mL by incorporating the surfactant (Tween 80) and water under controlled conditions.

Selection of Tween 80 as the surfactant and the formulation parameters used in this study were based on a previously established and validated method [33]. This approach allowed for investigating the impact of surfactant concentration on the stability and efficacy of the nanoemulsions while maintaining consistent levels of Thyme EO. Thyme EO was also prepared at the specified concentrations without undergoing the sonication process.

### Characterization of thyme essential oil nanoemulsion

The Characterization of TEON involved several analytical techniques to evaluate its physical and structural properties. Scanning Electron Microscopy (SEM) was used to observe the morphology and surface structure of the nanoemulsion particles. Particle size was measured using a Dynamic Light Scattering (DLS) analyzer at room temperature, which provided information on size distribution and uniformity. Zeta potential analysis was conducted to assess the stability and surface charge of the nanoemulsion, providing insights into electrostatic interactions and potential particle repulsion, both crucial for the stability and shelf-life of the formulation.

### Candida isolates and culture conditions

This study utilized 10 clinical isolates, including five of *C. glabrata* and five of *C. krusei*. These isolates were sourced from the Mycology Reference Center and Bacteriology Collection at the Faculty of Veterinary Medicine, University of Tehran, Tehran, Iran.

The *C. glabrata* and *C. krusei* isolates used in this study were previously identified and confirmed through both molecular methods and culture-based diagnostic tools [37] . These isolates have been preserved in the collection for subsequent analyses. Molecular identification of *C. glabrata* isolates was conducted, and their sequences were deposited in the National Center for Biotechnology Information (NCBI) database with accession numbers OR091292.1, OR091285.1, OR091282.1, OR091277.1, and OR091244.1.

For *C. krusei*, three isolates were molecularly confirmed with their sequences registered in the NCBI database under the accession numbers OR228911.1, MW819647.1, and MW714851.1. Molecular identification involved polymerase chain reaction (PCR) amplification of target regions, followed by sequencing and comparison with reference sequences in the NCBI database. Additionally, two other *C. krusei* isolates were identified using phenotypic methods. These isolates were cultured on Chromogenic Candida Agar (CHROMagar Candida, CHROMagar, Paris, France), a selective and differential medium that enables the rapid identification of *Candida* species based on colony color and morphology. The distinctive pink-to-purple coloration of *C. krusei* colonies on this medium provided a preliminary identification. Further confirmation was achieved using the RAPID Yeast Identification Kit (Remel, Thermo Fisher Scientific, Lenexa, KS, USA), which differentiates yeast species through a series of biochemical tests targeting enzymatic activity and metabolic profiles.

The confirmed isolates were cultured under controlled laboratory conditions to maintain viability and ensure experimental results were consistent. The isolates were routinely grown on Sabouraud Dextrose Agar (SDA) at 35 °C, a temperature conducive to the optimal growth of *Candida* species. For experiments, yeast cultures were subcultured into fresh media and incubated until they reached the appropriate growth phase, ensuring that the isolates were in a standardized state for subsequent testing and analysis. These standardized culture conditions were critical for reliable assessment of antifungal susceptibility and gene expression studies in this research.

### Antifungal activity

### Disk diffusion test

Antifungal activity of TEONE, Thyme EO, and FLZ on *Candida* isolates was evaluated using the disk diffusion method, following the Clinical and Laboratory Standards Institute (CLSI) M44 guidelines for testing antifungal susceptibility in yeasts [38].

Sterile Mueller-Hinton II agar plates supplemented with 2% glucose and 0.5 µg/mL methylene blue at a depth of 4.0 mm were inoculated with standardized suspensions of *C. glabrata* and *C. krusei* isolates, adjusted to a concentration of 1 × 10^6^ yeast/mL using a hemocytometer. Sterile blank paper discs were separately impregnated with the test substances, including TEONE, Thyme EO, and FLZ. The discs containing TEONE and Thyme EO were prepared at the desired concentrations, while the FLZ discs contained a standard concentration of 25 μg, serving as a reference antifungal agent. The impregnated discs were then placed onto the agar surface, ensuring adequate spacing to prevent overlapping of inhibition zones.

The plates were incubated at 35 °C for 24 h to allow yeast growth and the antifungal agents to diffuse from the discs into the agar medium. After the incubation period, the growth inhibition zones around each disc were measured using a caliper, with the diameter of clear zones indicating the antifungal activity of each substance. The results were interpreted based on the CLSI M44 standards, with larger inhibition zones corresponding to greater antifungal efficacy against the *Candida* isolates.

### Microdilution test

Antifungal activity of the tested agents was evaluated against *Candida* isolates using the micro-broth dilution method to determine the MIC and minimum fungicidal concentration (MFC) in accordance with the CLSI M27 guidelines for yeasts [39].

To perform the MIC test, two-fold serial dilutions of TEONE, Thyme EO, and FLZ were prepared in a 96-well microtiter plate, beginning at an initial concentration of 5,000 ppm. FLZ was similarly diluted with an initial concentration of 1 μg/mL. RPMI-1640 medium, buffered with MOPS (3-(N-morpholino) propanesulfonic acid) to a pH of 7.0, was used to create these dilutions. The final volume in each well was 100 µL, comprising 50 µL of the antifungal agent at varying concentrations and 50 µL of yeast inoculum. Each well was inoculated with a standardized suspension of *Candida* isolates, adjusted to a final concentration of approximately 1 × 10^3^ to 1 × 10^4^ yeast/mL [39]. To confirm growth, the positive control wells contained only Candida isolates without antifungal agents. The negative control wells contained only the culture medium without the presence of fungi or antifungal agents. In order to validate the test, the reference strain *Candida parapsilosis* ATCC 22019 was included as a control to ensure the accuracy and reproducibility of the MIC results.

The plates were incubated at 35 °C for 24 h. The MIC was defined as the lowest concentration of the antifungal agent that visibly inhibited the growth of the *Candida* isolates, as indicated by the absence of turbidity in the wells.

Following the MIC determination, the MFC was assessed by subculturing 10 μL from each well, showing no visible growth onto SDA plates. These plates were incubated at 35 °C for 24 h. The MFC was determined to be the lowest concentration of the antifungal agent, which resulted in no visible colony formation on the SDA plates, indicating fungicidal activity against the *Candida* isolates.

### Checkerboard assay

The interaction effect between TEONE and FLZ was investigated using the checkerboard method. This assay evaluated potential synergistic, additive, or antagonistic interactions between TEONE and FLZ against *Candida* isolates.

In this procedure, serial two-fold dilutions of TEONE (starting at concentrations twice the MIC values [μg/ml]) and FLZ (beginning at the initial concentration of 1 μg/mL) were prepared using RPMI-1640 medium, which was adjusted to a pH of 7.0. Dilutions of TEONE were prepared along the rows of a 96-well microtiter plate. In contrast, the dilutions of FLZ were prepared along the columns, creating a matrix of varying concentrations for each antifungal agent.

Each well in the microtiter plate contained a combination of different concentrations of TEONE and FLZ (50 µL), along with a standardized suspension of *Candida* isolates (50 µL), adjusted to a final concentration of approximately 1 × 10^3^ to 1 × 10^4^ yeast/mL. The plates were incubated at 35 °C for 24 h to allow for the interaction of the antifungal agents with the *Candida* isolates. The combined MIC was determined by selecting the well with the lowest concentration of both TEONE and FLZ, which exhibited complete inhibition of visible growth, as indicated by the absence of turbidity. This selection well represents the point of interaction between the two agents, ensuring accurate calculation of the fractional inhibitory concentration (FIC) and fractional inhibitory concentration index (FICI) values in accordance with standard checkerboard assay protocols. To validate the checkerboard assay, the reference strain *C. parapsilosis* ATCC 22019 was included as a control strain, following CLSI M27 guidelines, to confirm the reliability of the MIC and FICI values (μg/ml) [39].

The interaction effect was determined by measuring the growth inhibition in each well, with the results analyzed using the FICI. The FICI was calculated using the following formula:

$\mathrm{FICI}={\mathrm{FIC}}_{\mathrm{(TEONE)}}+{\mathrm{FIC}}_{\mathrm{(FLZ)}}$

Where FIC_(TEONE)_ = MIC of TEONE in combination / MIC of TEONE alone, and FIC_(FLZ)_ = MIC of FLZ in combination / MIC of FLZ alone. The FICI values were calculated to assess whether the combination of TEONE and FLZ exhibited synergistic (FICI≤0.5), additive (FICI>0.5 and ≤1.0), indifferent (FICI>1.0 and ≤4.0), or antagonistic (FICI>4.0) effects against the *Candida* isolates.

### Gene expression analysis

To investigate the expression of the *ERG11* gene in both isolates, as well as *CgCDR1* in *C. glabrata* and *ABC1* in *C. krusei*, the isolates were subjected to various treatments. The isolates were separately placed in the vicinity of different conditions; they were individually exposed to various conditions, including TEONE, Thyme EO, FLZ, a combination of TEONE and FLZ, and a combination of Thyme EO and FLZ.

The isolates were cultured at a concentration of 1 × 10^6^ yeast/mL in RPMI-1640 medium, maintaining a stable pH of 7.0 throughout the experiment. The exposure period involved incubating the treated isolates at 35 °C for a duration that allowed sufficient interaction between the cells and the antifungal agents, typically ranging from 24 h.

After the incubation period, total RNA was isolated from each sample using the RNA Isolation Kit from Sigma-Aldrich, following the protocol provided by the manufacturer. For the synthesis of complementary DNA (cDNA), the isolated RNA was reverse transcribed using the cDNA Synthesis Kit produced by Cinacloon, Iran, following the instructions of the manufacturer. Quantitative real-time PCR (qRT-PCR) was performed to determine the expression levels of the target genes (*ABC1*, *CgCDR1*, and *ERG11*) relative to a housekeeping gene, *ACT1* (Table 1). The qRT-PCR process was conducted following the method outlined by Paul et al., using a Light Cycler 480 system (Roche, Switzerland) [40]. For each RT-qPCR experiment, two negative controls were included: one containing all components except cDNA and another lacking primer to ensure the accuracy and reliability of the results. The relative expression levels of the resistance genes were then analyzed to assess the impact of TEONE, Thyme EO, FLZ, and their combinations on antifungal resistance mechanisms in *Candida* isolates.

**Table 1:** Primer sequences for genes used in quantitative real-time polymerase chain reaction.

Target Gene	Primer Type	Sequence (5' - 3')	Reference
*ABC1*	Forward	GATAACCATTTCCCACATTTGAGT	[41]
	Reverse	CATATGTTGCCATGTACACTTCTG	
*CgCDR1*	Forward	CATACAAGAAACACCAAAGTCGGT	[42]
	Reverse	GAGACACGCTAACGTTCACCAC	
*ERG11*	Forward	ACTAGATGGGATACTGCTGC	[43]
	Reverse	CATCTATGTCTACCACCACC	
*ACT1*	Forward	ACTACCATGTTCCCAGGTATTG	[43]
	Reverse	CCACCAATCCAGACAGAGTATT	

### Statistical analysis

All experiments were conducted in triplicate to ensure the accuracy and reliability of the results. Data from the antibacterial study were expressed as mean ± standard error of the mean (SEM) due to the repeated variability observed across measurements. In contrast, the particle size measurements were reported as mean ± standard deviation SD) to reflect the dispersion of particle sizes from the average. Statistical analysis was performed using GraphPad Prism (version 10.3.0) software. ANOVA was used to compare the means of different groups, followed by post hoc tests to determine significant differences. A p-value of less than 0.05 was considered statistically significant. For gene expression data, relative quantification was calculated using the 2^−ΔΔCt^ method [44], and statistical significance was assessed using t-tests to compare treated groups with controls. Gene expression data were analyzed using StepOne^TM^ Software v2.3 (Applied Biosystems), employing the comparative Ct (ΔΔCt) method for quantifying relative changes in target gene expression.

## Results

### Characterization of thyme essential oil nanoemulsion

The SEM images indicated that the nanoemulsion predominantly exhibited a spherical morphology, with only a minimal presence of rod-shaped particles. Importantly, no significant accumulation of nanoparticles was observed, suggesting a well-dispersed formulation. The largest nanoparticle recorded size was 40.73 nm, confirming the small-scale nature of the particles (Figure 1).

**Figure 1:**
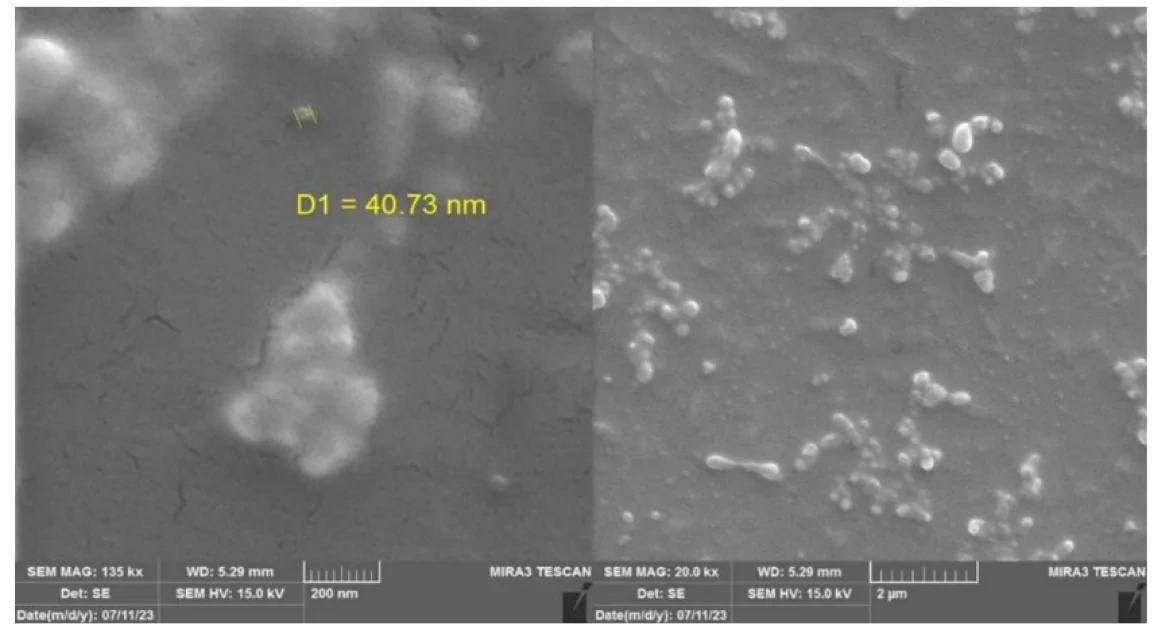
Scanning Electron Microscopy images of nanoemulsion particles (thyme essential oil nanoemulsion ). The images show predominantly spherical morphology with minimal rod-shaped particles. The largest observed nanoparticle size is 40.73 nm, indicating a well-dispersed formulation without significant nanoparticle aggregation.

DLS analysis further characterized the formulation, showing a mean droplet diameter of 134.3 nm with an SD of 48.7 nm. The surface-to-particle area (S.P.Area) ratio was one, indicating a consistent particle distribution. The polydispersity index (PI) was measured at 0.402, highlighting a suitable dispersion quality and confirming that the nanoemulsion is polydisperse. The Z-average, which provides an intensity-weighted mean hydrodynamic diameter, was calculated to be 126.3 nm (Figure 2). These findings from SEM are consistent with the size measurements provided by DLS analysis, reinforcing the reliability of the results.

**Figure 2:**
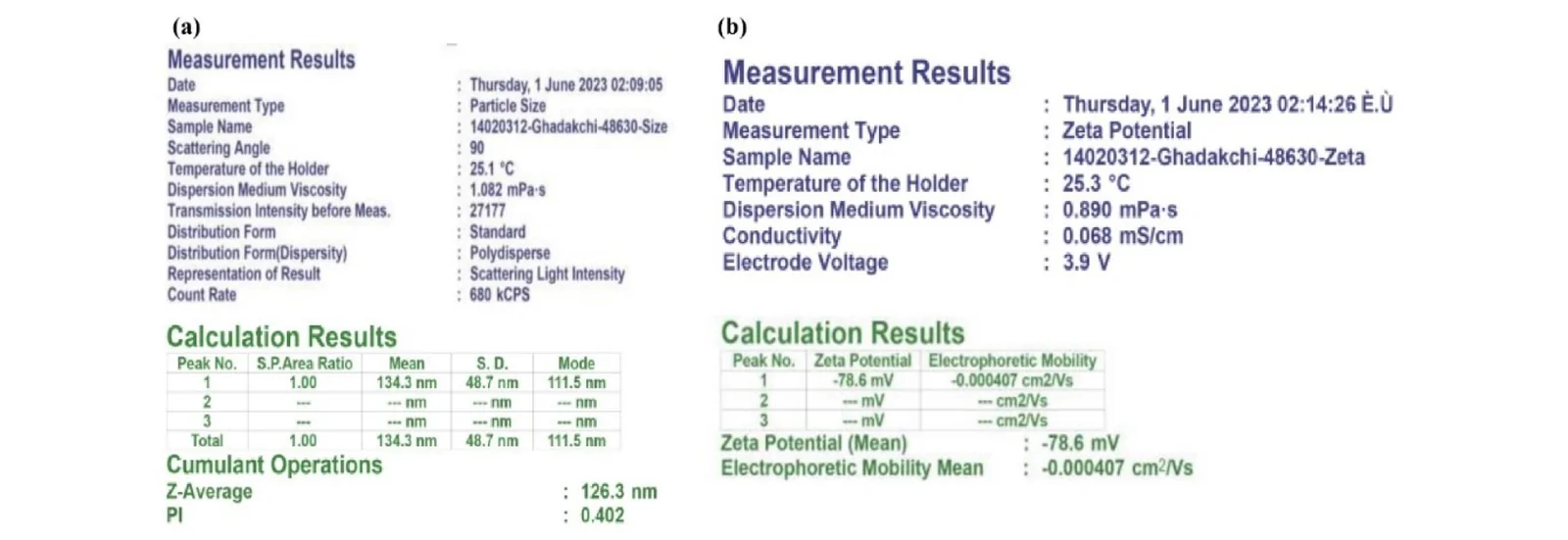
(**a**) Dynamic Light Scattering analysis results show the particle size distribution of the nanoemulsion with a Z-average of 126.3 nm and a polydispersity index of 0.402. (**b**) Zeta Potential analysis results indicate the surface charge stability of nanoemulsion with a mean Zeta Potential value of -78.6 mV and an electrophoretic mobility of -0.000407 cm^2^/Vs.

Zeta potential analysis, which assesses the stability of nanoemulsion by measuring the surface charge of particles, showed a mean zeta potential of -78.6 mV. This highly negative value suggests good stability due to strong electrostatic repulsion between particles, reducing the likelihood of aggregation. Additionally, the mean electrophoretic mobility was measured at -0.000407 cm^2^/Vs, supporting the conclusion of a stable and well-dispersed nanoemulsion system (Figure 2).

### Disk diffusion test results

The disk diffusion test results demonstrated that TEONE and Thyme EO exhibited notable antifungal activity against *C. glabrata* and *C. krusei*. Overall, TEONE showed superior antifungal effects compared to Thyme EO across the tested concentrations, indicating the enhanced efficacy of the nanoemulsion formulation. No significant differences were found in the effects of the antifungal agents across the different *Candida* isolates. The variation in the effects of antifungal agents between isolates within each species was insignificant.

The TEONE demonstrated significant growth inhibition zones (*p*<0.01), especially at higher concentrations, suggesting potent antifungal properties against both *Candida* species. For example, at 5,000 ppm, TEONE consistently produced large inhibition zones, reflecting its potent efficacy. Even at lower concentrations, such as 2,500 ppm, TEONE maintained substantial inhibition, indicating its effectiveness over various concentrations. While effective, Thyme EO generally showed smaller inhibition zones than TEONE at equivalent concentrations. Activity of Thyme EO decreased more noticeably at lower concentrations, demonstrating less consistent antifungal action across the concentration gradient (at concentrations below 2,000 ppm). Despite this, Thyme EO still exhibited meaningful antifungal activity, particularly at higher concentrations (Figure 3). Interestingly, all the *Candida* isolates tested in this study resisted FLZ.

**Figure 3:**
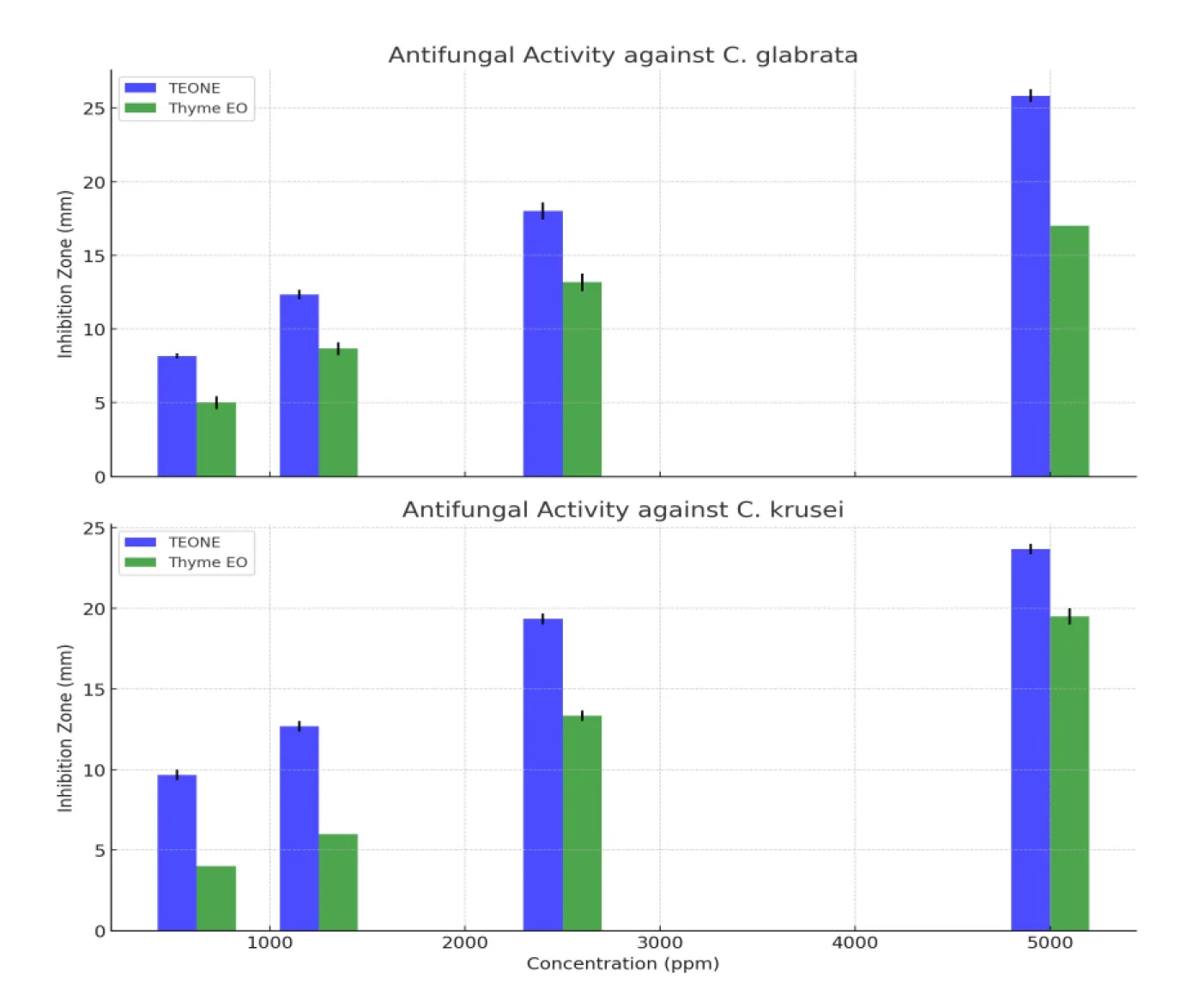
Antifungal activity of thyme essential oil nanoemulsion (TEONE) and thyme essential oil (thyme EO) against *Candida glabrata* and *Candida krusei* was measured by a disk diffusion test. Error bars indicate the Scanning Electron Microscopy. The inhibition zones (mm) are shown across various concentrations (5,000 ppm, 2,500 ppm, 1,250 ppm, and 625 ppm). The TEONE consistently exhibits larger inhibition zones, compared to Thyme EO, indicating its superior antifungal efficacy, especially at higher concentrations. The growth inhibition zone for each species is taken as an average value.

### Microdilution Test Results

The microdilution test results highlighted the superior antifungal efficacy of TEONE, compared to traditional Thyme EO against *C. krusei* and *C. glabrata* isolates (*p*<0.05). The TEONE consistently demonstrated lower MIC and MFC values (μg/ml) across all tested isolates. In particular, *C. glabrata* isolates showed a remarkably high sensitivity to TEONE, requiring significantly lower concentrations to achieve both inhibitory and fungicidal effects. This suggests that the nanoemulsion formulation enhances the bioavailability and efficacy of Thyme EO, making TEONE a more effective treatment option.

Statistical analysis further validated these findings, showing a significant difference in the efficacy of TEONE, compared to Thyme EO (*p*<0.05). For *C. krusei*, the mean MIC for TEONE was approximately 437.5 ppm, while the mean MIC for Thyme EO was significantly higher at around 1,600 ppm. For *C. glabrata*, TEONE demonstrated even greater efficacy, with a mean MIC of approximately 156.25 ppm, compared to 875 ppm for Thyme EO. The standard deviation for MIC and MFC values was relatively low for TEONE, indicating consistent antifungal activity across different isolates. At the same time, the higher variability observed with Thyme EO suggests differences in isolate susceptibility or lower overall efficacy (Figure 4).

**Figure 4:**
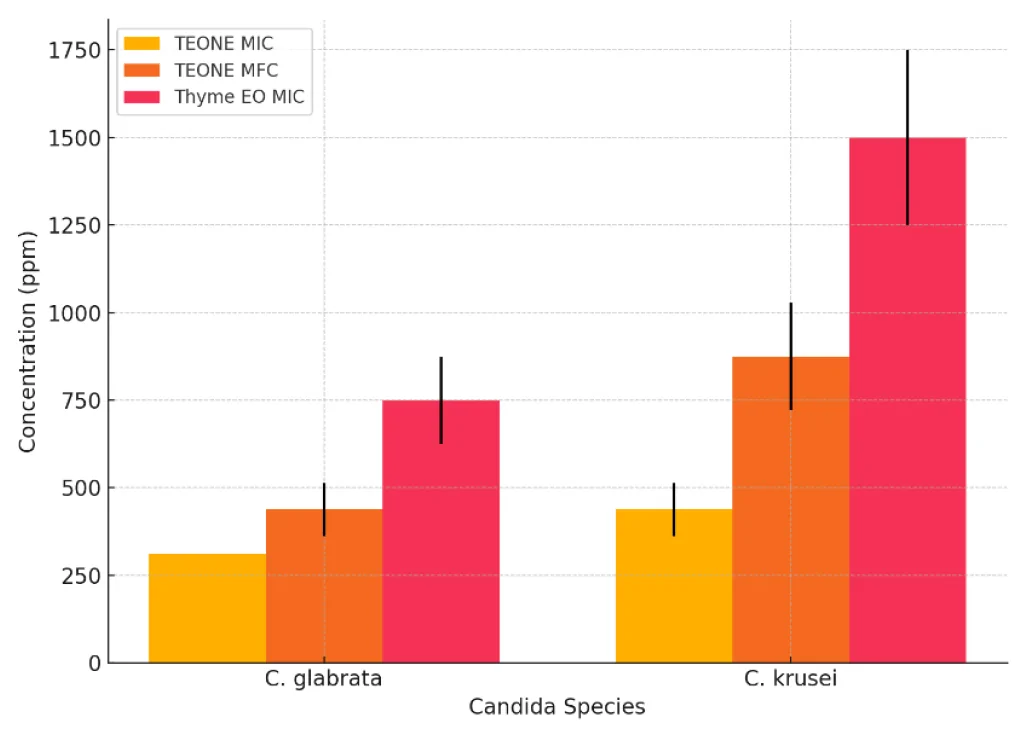
Minimum inhibitory concentration and minimum fungicidal concentration of thyme essential oil nanoemulsion and thyme essential oil against *Candida glabrata* and *Candida krusei*. Bars represent the mean values, with error bars indicating the standard error of the mean. *Candida krusei* exhibited higher minimum inhibitory concentration and minimum fungicidal concentration values, compared to *C. glabrata*, suggesting reduced susceptibility to both agents.

### Checkerboard assay results

Findings of the assay consistently showed an additive interaction between TEONE and FLZ across all tested isolates. For *C. krusei* isolates, the FIC values for TEONE ranged from 0.3 to 0.5, while the FIC values for FLZ varied between 0.47 and 1.0. The resulting FICI values for *C. krusei* ranged from 0.55 to 0.95, indicating a clear additive effect. Similarly, for *C. glabrata* isolates, the FIC values for TEONE ranged from 0.25 to 0.5, and the FIC values for FLZ varied between 0.25 and 2.0, resulting in FICI values between 0.55 and 0.95. These results confirm that the combination of TEONE and FLZ consistently produced an additive effect (FICI>0.5 and≤1.0) across all isolates, improving antifungal efficacy without achieving a synergistic interaction (Table 2).

**Table 2:** Checkerboard assay results.

*Candida* isolates	Combined MIC (μg/ml)		FIC (μg/ml)		FICI	Interpretation
FLZ	TEONE	FLZ	TEONE			
*C krusei* 1	64	312.5	0.47	0.3	0.55	Additive
*C. krusei* 2	64	312.5	1	0.47	0.65	Additive
*C. krusei* 3	64	625	1	0.5	0.75	Additive
*C. krusei* 4	64	312.5	1	0.5	0.85	Additive
*C. krusei* 5	64	312.5	0.5	0.5	0.95	Additive
*C. glabrata* 1	64	156.25	1	0.5	0.55	Additive
*C. glabrata* 2	32	156.25	0.5	0.25	0.65	Additive
*C. glabrata* 3	64	312.5	2	0.5	0.75	Additive
*C. glabrata* 4	32	156.25	0.5	0.25	0.85	Additive
*C. glabrata* 5	32	156.25	1	0.25	0.95	Additive

FIC: fractional inhibitory concentration, FLZ: fluconazole, TEONE: thyme essential oil nanoemulsion, FICI: fractional inhibitory concentration index

### Impact on resistance gene expression

The qRT-PCR analysis revealed significant changes in the expression of antifungal resistance genes *ERG11*, *ABC1*, and *CgCDR1* in *C. krusei* and *C. glabrata* isolates in response to different treatments (Figure 5). Among the antifungal agents tested, TEONE was the most effective in reducing gene expression across both isolates.

In *C. krusei*, treatment with TEONE caused a notable decrease in *ERG11* expression, reducing it by approximately 49% (fold change of 0.51). Thyme EO further reduced *ERG11* expression, resulting in a 45% reduction (fold change of 0.55). In contrast, FLZ treatment significantly upregulated *ERG11* expression by around 36% (fold change of 1.36). When TEONE and FLZ were combined, *ERG11* expression decreased by 15% (fold change of 0.85), compared to FLZ alone. A combination of Thyme EO and FLZ led to a slight reduction in expression levels by 9% (fold change of 0.91). In *C. glabrata*, TEONE treatment led to an 18% reduction in ERG11 expression (fold change of 0.82). Thyme EO had a negligible impact, resulting in a 6% decrease (fold change of 0.94). FLZ significantly elevated *ERG11* expression by about 71% (fold change of 1.71). The combination treatments, TEONE + FLZ and Thyme EO + FLZ, showed moderated effects, with a 4% increase (fold change of 1.04) and a 5% increase (fold change of 1.05) in *ERG11* expression, respectively. However, given the minimal magnitude of change, these effects may lack biological significance and should be interpreted with caution.

For the *ABC1* gene in *C. krusei*, TEONE caused a significant decrease in expression, reducing it by approximately 51% (fold change of 0.49). Thyme EO also decreased *ABC1* expression by 14% (fold change of 0.86). In contrast, FLZ markedly increased *ABC1* expression by 66% (fold change of 1.66). When TEONE was combined with FLZ, *ABC1* expression was reduced by 24% (fold change of 0.76), compared to FLZ alone. Similarly, combining Thyme EO with FLZ led to a modest decrease of 5% (fold change of 0.95).

Regarding *CgCDR1* expression in *C. glabrata*, TEONE significantly reduced expression by 49% (fold change of 0.51). Thyme EO also decreased *CgCDR1* expression, but to a lesser extent, with a reduction of 29% (fold change of 0.71). FLZ treatment, on the other hand, significantly increased *CgCDR1* expression by 95% (fold change of 1.95). Combination treatments with TEONE and FLZ moderated this expression level, showing a reduction of 22% (fold change of 0.78). Combination of Thyme EO with FLZ further decreased *CgCDR1* expression by 27% (fold change of 0.73).

Between the two isolates, *C. glabrata* exhibited a more pronounced reduction in gene expression in response to the antifungal agents, particularly TEONE. This isolate demonstrated a marked downregulation of *CgCDR1* and *ERG11*, suggesting that *C. glabrata* is more susceptible to the inhibitory effects of TEONE. In contrast, *C. krusei* showed resistance patterns, especially when treated with FLZ, which resulted in an upregulation of *ERG11* and *ABC1*. Among the genes analyzed, *CgCDR1* in *C. glabrata* was the most affected by the antifungal treatments.

**Figure 5:**
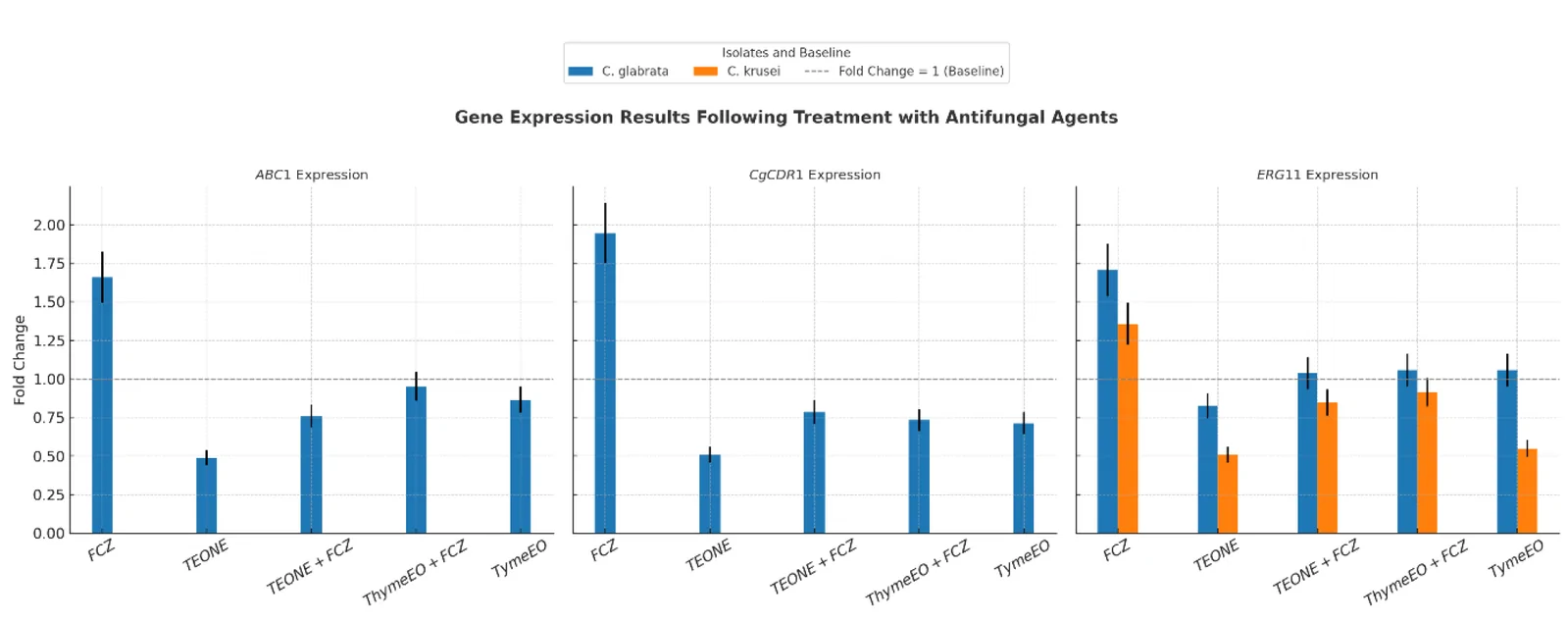
Fold change in the expression of *ABC1*, *CgCDR1*, and *ERG11* genes in *Candida glabrata* and *Candida krusei* isolates following treatment with antifungal agents. Error bars represent the standard error of the mean calculated from three experimental replicates. The dashed line at fold change = 1 indicates the baseline expression level. Thyme essential oil nanoemulsion (TEONE) and its combinations significantly reduced gene expression, compared to FLZ alone, demonstrating their potential to mitigate antifungal resistance.

## Discussion

Exploration of TEONE and Thyme EO against FLZ-resistant Candida species, particularly *C. krusei* and *C. glabrata*, represents an innovative direction in antifungal research. Results of this study provide valuable insights into the efficacy of TEONE, compared to traditional Thyme EO and FLZ, in managing *Candida* infections. The findings highlight the potential of TEONE, both as a standalone treatment and in combination with FLZ, to enhance antifungal efficacy and reduce the expression of crucial resistance genes in *C. krusei* and *C. glabrata*. The TEONE consistently achieved larger inhibition zones and lower MIC and MFC values, indicating its superior ability to inhibit and kill these *Candida* species.

Furthermore, the combination of TEONE with FLZ showed additive effects, suggesting enhanced antifungal activity without antagonism, potentially allowing for lower doses of FLZ to be used effectively. The qRT-PCR analysis revealed that TEONE significantly downregulated the expression of crucial resistance genes (*ABC1*, *CgCDR1*, and *ERG11*) in both *C. krusei* and *C. glabrata*, while FLZ alone often upregulated these genes. These findings highlight the potential of TEONE, both alone and in combination with FLZ, to manage antifungal resistance and improve the efficacy of current treatments against *Candida* infections.

Findings of the current study are in line with those of Hassanin et al. (2023) regarding the potential of TEONE as a powerful antifungal agent capable of effectively managing fungal infections across different contexts, whether in agricultural or human health contexts. The enhanced antifungal activity observed with TEONE against *Candida* species and *Sclerotinia sclerotiorum* can be attributed to the improved delivery and efficacy of the active compounds, such as thymol, 1,8-cineole, α-terpinene, and ρ-cymene, as evidenced by gas chromatography analysis. The smaller particle size of nanoemulsion, as observed in both studies (36.65 nm in the study performed by Hassanin et al. and similarly small sizes in the present study using DLS analysis), is likely to play a crucial role in enhancing its bioavailability and penetration into fungal cells, leading to more effective inhibition of growth and downregulation of resistance genes [45]. Hashem et al. reported that their clove and TEONE (CL+TH-nanoemulsion) achieved higher antimicrobial and antibiofilm activities, compared to the non-nanoemulsified form. The smaller droplet size of nanoemulsion (68.6 nm) and improved stability likely contributed to its enhanced antimicrobial effects [34].

It is important to address the apparent discrepancy between the nanoparticle size measurements obtained via SEM (maximum 40.73 nm) and DLS (Z-average 126.3 nm). This divergence stems from fundamental differences in analytical methodologies. SEM provides the actual physical size of dehydrated particles under high vacuum, while DLS measures the hydrodynamic diameter, which includes the core particle, its solvation layer, and any associated surfactant shell [46, 47]. Moreover, because DLS is an intensity-weighted technique, it tends to emphasize larger particles or aggregates within polydisperse systems, resulting in higher reported average sizes. These differences are not contradictory but complementary, offering a more complete understanding of the behavior of nanoemulsion under both dry and hydrated conditions. Recognizing these distinctions is essential for interpreting particle size in the context of biological efficacy and formulation stability [47-49].

Findings of the present study, in conjunction with the results reported by Liu et al. (2024), emphasize the significant role that nanoemulsion formulations play in enhancing the antifungal efficacy of natural compounds. Inhibition of *Candida* species growth by TEONE in the current study reflects the findings reported by Liu et al., where a eugenol nanoemulsion containing Nobiletin (EGN) showed superior antifungal activity, compared to pure eugenol nanoemulsion, against *Penicillium italicum*, achieving a lower MIC and a higher rate of mycelial growth inhibition. Both studies underscore the potential of nanoemulsions to improve the bioavailability and penetration of antifungal compounds, thereby enhancing their effectiveness.

The observed ability of TEONE to downregulate resistance genes in *Candida* species aligns with the findings from Liu et al., where EGN caused more significant structural damage to fungal cells than its non-nano-emulsified counterpart. This suggests that nanoemulsions improve antifungal efficacy and offer a mechanism to overcome resistance and enhance the integrity of antifungal treatments [50].

The findings from the present study, along with those reported by Nikkhah et al. (2024), demonstrate the effectiveness of nanoemulsion-based formulations in boosting the antifungal properties of natural agents. Results of the current research are in line with those of a study performed by Nikkhah et al., where a nanoemulsion combining crude Reuterin with synergistic EO showed potent antifungal effects against various fungal pathogens, including *Botrytis cinerea*, *Penicillium expansum*, and *Alternaria alternata*. The optimized nanoemulsion, as reported by Nikkhah et al., also displayed excellent stability and a reduced particle size, enhancing its antifungal effectiveness and capability to protect apples from spoilage [51].

A study carried out by Al-Asmari et al. showed similarities with the current research, as both TEONE and origanum EO nanoemulsion demonstrated significantly enhanced antimicrobial effectiveness, compared to their non-nanoemulsified forms. The ability of TEONE to substantially reduce microbial populations, achieving a 5-log reduction in *Escherichia coli* and a 4-log reduction in *Saccharomyces cerevisiae*, underscores its potent antimicrobial capabilities. Both studies highlight the critical role of nanoemulsion parameters, such as droplet size, surfactant concentration, and hydrophilic-lipophilic balance, in optimizing the stability and efficacy of the formulations. The smaller droplet sizes achieved in TEONE and TEON formulations facilitate more excellent surface area contact with microbial cells, improving the penetration and activity of the EO components. Moreover, the stability of nanoemulsion, indicated by the lack of phase separation, ensures consistent antimicrobial performance, making these formulations highly effective [52].

Similar to the results of the current investigation, Adisuri et al. found that a Piperine (black pepper)-loaded nanoemulsion substantially enhanced antifungal activity, compared to pure Piperine in DMSO, showing lower MIC_90_ values across multiple *Candida* species. The increased bioavailability and effectiveness of both TEONE and the Piperine-loaded nanoemulsion can be attributed to their smaller droplet sizes and improved stability. Adisuri et al. reported that their nanoemulsion formulation had significantly lower MIC_90_ values than pure Piperine in DMSO, with reductions ranging from approximately 20–50% across various *Candida* strains. Furthermore, the Piperine-loaded nanoemulsion demonstrated notable stability and bioavailability, characterized by a small droplet size (24.37 nm), a polydispersity index of 0.453, and a zeta potential of -21.10 mV [53].

Several studies have demonstrated that changes in mitochondrial functions play a critical role in the susceptibility of fungal cells to various antifungal molecules [54]. Mitochondria, as essential organelles in cellular energy production and metabolism, have emerged as important targets for antifungal drug development [55, 56]. Desired effects of TEONE in the present study can be attributed to the production of reactive oxygen species (ROS) when the cell is exposed to the active ingredients in thyme EO, including its major compound, thymol. It should be noted that in the nano-release and retention state, these active ingredients are present at concentrations several times higher than in their normal state [28].

The ROS are produced in mitochondria when cells are exposed to antifungal compounds or phytocompound mixtures, leading to oxidative damage to key cellular structures, such as the cell membrane, proteins, lipids, and nucleic acids. Additionally, ROS can activate pro-apoptotic signals, contributing to cell death [56]. These effects suggest that mitochondrial dysfunction and ROS production are closely linked to the antifungal activity of compounds, such as thymol and FLZ, reinforcing the notion that mitochondria may be a promising target for developing novel antifungal therapies.

The current research demonstrated that TEONE effectively downregulated key resistance genes, thereby enhancing the treatment efficacy against infections caused by *C. krusei* and *C. glabrata*, especially when combined with FLZ. Conversely, using FLZ alone frequently led to the upregulation of these resistance genes, suggesting a potentially counterproductive effect when utilized as a monotherapy [57]. Notably, the combination of TEONE with FLZ led to a moderation in gene expression levels, suggesting that TEONE can help mitigate FLZ-induced resistance mechanisms. This ability to downregulate resistance genes makes TEONE a valuable option for overcoming the limitations of current antifungal therapies and managing drug-resistant *Candida* infections. Findings of the current study are consistent with those reported by Parsameher et al., who reported that biogenic selenium nanoparticles exhibited significant antifungal effects against both azole-resistant and wild-type *Candida albicans* strains. Selenium nanoparticles inhibited fungal growth at 100 µg/mL concentrations for resistant strains and 70 µg/mL for susceptible strains. Similarly, they downregulated the expression of *CgCDR1* and *ERG11* genes, which are associated with azole resistance. Both studies highlight the potential of nano-based formulations to enhance antifungal efficacy and overcome resistance mechanisms by targeting specific genetic pathways involved in drug resistance [36].

The present investigation explored the impact of various antifungal agents, with a particular focus on the role of EO nanoemulsions in modulating antifungal resistance. Studies have shown that novel agents, such as selenium nanoparticles and EO nanoemulsions, can effectively downregulate the expression of *ABC1*, *CDR1*, and *ERG11*, reducing resistance and enhancing antifungal efficacy [36]. In the current study, TEONE has emerged as a highly effective antifungal agent, significantly reducing resistance gene expression in both *C. glabrata* and *C. krusei*. In contrast, Thyme EO demonstrated moderate efficacy, particularly against *ERG11* and *ABC1* in *C. krusei*. The nanoemulsion formulation enhances the structural integrity, bioactivity, contact surface, stability, and effectiveness of Thyme EO, making it more effective than traditional EO [58].

It is noteworthy that FLZ generally increased gene expression, particularly upregulating resistance genes, such as *CgCDR1*, in *C. glabrata*, indicating its role in potentially inducing antifungal resistance mechanisms. Role of FLZ in increasing resistance gene expression highlights the need for alternative antifungal strategies similar to TEONE. Findings of this investigation highlight the potential of EO nanoemulsions, especially TEONE, as promising alternatives to conventional antifungal agents, such as FLZ, which can induce resistance. By effectively reducing the expression of key resistance genes, such as *ABC1*, *CDR1*, and *ERG11*, EO nanoemulsions could offer a more efficient strategy for combating antifungal resistance, enhancing the efficacy of antifungal treatments, and addressing the growing concern of drug-resistant fungal infections.

Results of the present research regarding gene expression are consistent with those of a study performed by Lamping et al., who identified the natural resistance of *C. krusei* to FLZ as being primarily due to the low affinity of Lanosterol 14-α-demethylase (Erg11p) for the antifungal drug and the continuous expression of the multidrug efflux pump Abc1p. Lamping et al. demonstrated that the presence of multiple *ERG11* alleles, along with the constitutive expression of *ABC1* contributed to high levels of azole resistance in *C. krusei*. Both studies highlight the critical role of efflux pumps and the *ERG11* gene in mediating azole resistance. However, while Lamping et al. showed that the innate resistance in *C. krusei* is due to these factors, the current study revealed that nanoemulsions, like TEONE, can counteract these resistance mechanisms by downregulating the expression of *ABC1* and *ERG11*, thereby enhancing the efficacy of antifungal treatments [13].

Combination of TEONE and FLZ resulted in alterations in mitochondrial function and nuclear morphology, indicating a disruption of cellular integrity in resistant strains [54]. Ricardo et al. observed that *C. krusei* could develop resistance to VRC during prolonged exposure, both *in vivo* and *in vitro*. Ricardo et al. noted that resistance was associated with increased *ABC1* gene expression and specific mutations in the *ERG11* gene, highlighting the role of efflux pumps and genetic mutations in mediating azole resistance. The synergistic effect observed between efflux pump inhibitors, such as tacrolimus (FK506), and VRC in resistant strains further supports the critical role of the Abc1p efflux pump in drug resistance. Similar to these findings, the current investigation illustrated that the downregulation of *ABC1* and *ERG11* gene expression by TEONE can reverse resistance mechanisms, offering a viable strategy to combat azole-resistant *Candida* infections [59].

Novelty of the present study lay in its unique approach of combining TEONE with conventional antifungal treatment to tackle drug-resistant *Candida* species, particularly *C. krusei* and *C. glabrata*. Unlike previous studies that focused solely on natural or conventional antifungal agents, this study highlighted the additive potential of nanoemulsions to enhance the efficacy of existing drugs, such as FLZ. Additionally, this research provided a comprehensive analysis of how TEONE impacts the expression of critical resistance genes, offering valuable insights into its mechanism of action against resistant *Candida* strains. This innovative combination of nanoemulsion technology with conventional antifungal therapy represents a promising strategy to improve treatment outcomes for difficult-to-treat *Candida* infections. It addresses the growing concern of antifungal resistance.

Physicochemical characterization of nanoemulsions is essential for their use in antifungal treatments, as properties, such as particle size and PI, significantly affect their stability and effectiveness. Nanoemulsions typically fall within a particle size range of 70-240 nm, which is critical for optimal bioavailability and stability [22]. In the current study, the nanoemulsion formulation exhibited a particle size within this acceptable range and a PI value of less than one, indicating a high degree of uniformity and stability suitable for antifungal applications. A PI value below one signifies uniformity, crucial for consistent antifungal efficacy, including Tween 80 as a surfactant facilitated rapid adsorption onto the droplet surface, reducing droplet diameter and enhancing the stability and effectiveness of the nanoemulsion. This size reduction improves stability and enhances bioactivity, as demonstrated in a study conducted by Nikkhah et al., where the smallest particle size achieved was 16.3 nm using similar methods [51]. These findings confirm that the nanoemulsion formulation meets the essential criteria for effective antifungal activity, supporting its potential for use in clinical settings.

## Conclusion

Findings of the present study highlighted the promise of TEONE as a potent antifungal agent capable of downregulating the expression of resistance genes and boosting the efficacy of conventional treatments, such as FLZ. These findings underscore the broader potential of nanoemulsion-based formulations in enhancing the delivery and bioavailability of natural antifungal agents, reducing drug resistance, and improving therapeutic outcomes.

The approach employed in the present study can help lower the doses of antifungals like FLZ, thereby minimizing the risk of drug resistance. Usage of nanoemulsion technology to deliver Thyme EO components offers a novel approach to overcoming resistance in *Candida* species, potentially improving clinical outcomes in patients suffering from fungal infections. The findings advocate for further exploration of TEONE in combination therapies to combat the growing challenge of antifungal resistance in clinical settings.
